# Mobile Virtual Learning Object for the Assessment of Acute Pain as a Learning Tool to Assess Acute Pain in Nursing: An Analysis of the Mental Workload

**DOI:** 10.2196/mededu.4958

**Published:** 2015-11-06

**Authors:** Ana Graziela Alvarez, Grace Sasso, Sriram Iyengar

**Affiliations:** ^1^ Science Health Center Department of Nursing Federal University of Santa Catarina Florianópolis Brazil; ^2^ Texas A&M University Biomedical Informatics Core, Clinical Science and Translational Research University of Texas Houston, TX United States

**Keywords:** nursing, nursing informatics, distance learning, computer-assisted instruction, educational technology, nursing education, acute pain, persuasive technology, mental workload

## Abstract

**Background:**

The inclusion of new technologies in education has motivated the development of studies on mental workload. These technologies are now being used in the teaching and learning process. The analysis enables identification of factors intervening in this workload as well as planning of overload prevention for educational activities using these technologies.

**Objective:**

To analyze the mental workload of an educational intervention with the Mobile Virtual Learning Object for the Assessment of Acute Pain in adults and newborns, according to the NASA Task Load Index criteria.

**Methods:**

A methodological study with data collected from 5 nurses and 75 students, from November of 2013 to February of 2014.

**Results:**

The highest students’ and specialists’ means were in the dimensions of “Mental demand” (57.20 ± 22.27; 51 ± 29.45) and “Performance” (58.47 ± 24.19; 73 ± 28.85). The specialists’ mental workload index was higher (50.20 ± 7.28) when compared with students’ (47.87 ± 16.85) on a scale from 0 to 100 (*P*=.557).

**Conclusions:**

The instrument allowed for the assessment of mental workload after an online educational intervention with a mobile learning virtual object. An excessive overload was not identified among participants. Assessing mental workload from the use of educational technologies at the end of a task is a key to their applicability, with the aim of providing a more effective, stimulating, and long-lasting experience of the learning process.

## Introduction

Following the evolution of information and communications technologies (ICTs), the concept of the virtual learning object (VLO) has been emphasized in the educational scenario. It is defined as a unit that creates an educational context, is reusable in different learning contexts, and consists of an active and constructive teaching-learning strategy [1-3].

The interest in research on the organization of mental workload has gradually increased given the popularization of information and communications technologies (ICTs) in the workplace. The use of technology has also increased in teaching and learning, resulting in a growing interest for understanding the flow of information, as well as the mental workload generated by the use of these technologies [4].

Considering the current technological advances, some issues related to the teaching and learning process need to be reviewed; additionally, there is a need to establish metrics for the effective assessment of important parameters that can affect students’ performance, such as mental workload.

Mental workload can be defined as the interaction between internal and external factors and the individual, resulting in a subjectively described experience [5]. This is the effect that a particular demand has on the individual in terms of mental and physical effort, which can be related to the amount of processed information, as well as the effort for the performance of that particular task [6].

It should be noted that the assessment of mental workload is subjective, namely, it is assessed from the perspective of the person performing the task, not the task itself. The assessment depends on individual characteristics that can increase or decrease this individual perception. These characteristics include the individual’s knowledge for performing the task, experience with the activity, age, education, humor, etc. [7-9].

The assessment of mental workload assumes that an individual’s burnout level is directly associated with his/her ability to perform a certain activity [10-12]. In this sense, the NASA Task Load Index (NASA TLX) has been more widely used, which is freely available to researchers (see Multimedia Appendix 1).

Although the interest in measuring mental workload as a means of understanding human-machine interfaces has been around for some years, only for the last 20 years has there been interest in the study of this effect on Web-based learning in humans [13-19].

By identifying the rates of the dimensions that construct the mental workload, it can be classified as either “overloaded” or “underloaded.” Overload corresponds to a saturation of resource consumption, whereas underload results from the lack of stimulus to perform the task [20].

The calculation of mental workload through the NASA TLX combines the assessment rates of different dimensions and their weights, weighted according to their subjective importance for a given task [21]. In addition, studying the factors intervening in mental workload from a given task enables the planning of overload prevention during activities performed by these individuals [20], and can assist with the planning and developing of educational technologies.

The aim of this study was to analyze the mental workload of an educational intervention using the Mobile Virtual Learning Object for the Assessment of Acute Pain (m-OVADor) in adults and newborns, according to the NASA TLX criteria.

## Methods

A methodological study [22] approved by the Research Ethics Committee at the Universidade Federal de Santa Catarina (UFSC) in 2012 (certificate 2456), with online data collection from November 1/2013 to February 15/2014.

A total of 170 students in the second to the eighth semesters of the undergraduate nursing course were eligible to participate in this study, of which 120 agreed to do so. The nonprobabilistic and intentional final sample, after applying the exclusion criteria, included 75 students and 5 specialist nurses, who completed all planned steps. All participants were identified by alphanumeric codes, students S1 to S75 and nurse specialists NS1 to NS5.

The inclusion criteria for students were (1) being regularly enrolled in the second to the eighth semester of the undergraduate nursing course at UFSC during the study period and (2) being available for online participation in the study during an extracurricular period. The inclusion criteria for nurse specialists were (1) minimum of a master’s degree; (2) a nurse with minimum experience of 2 years in the critical care area, neonatology/pediatrics, and/or medical-surgical clinic; (3) experience with the use or development of ICTs in the teaching-learning process in nursing; (4) having a mobile device with Internet access (cell phone, smartphone, personal digital assistant, or tablet). The exclusion criterion was missing any of the study steps.

All participants received the instructions to participate in the study via email and Facebook. The m-OVADor was accessed from their own mobile devices (see Multimedia Appendix 2).

**Figure 1:**
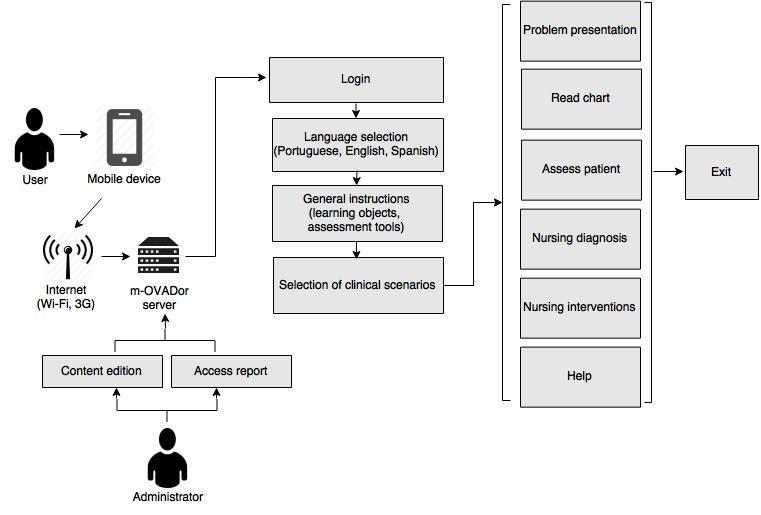
m-OVADor structure for access and assessment of acute pain in clinical scenarios.

The technology developed for the study had 3 different simulated clinical scenarios (surgical clinic, adult intensive care, and neonatology; see Multimedia Appendix 3), which enabled the study of different variables involved in the assessment of acute pain (Figure 1).

**Figure 2:**
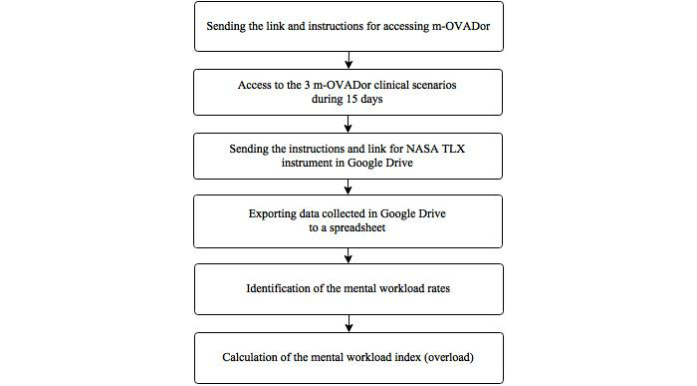
Protocol for the analysis of mental workload from the NASA TLX instrument.

Identification of the measures required for the analysis of mental workload of students and specialists (rate, weight, magnitude, and mental workload index) was performed by application of the NASA TLX instrument [11], according to the following protocol (Figure 2).

The NASA TLX used in the study is a version that was translated and adapted by the authors, available for free use on the NASA website (see Multimedia Appendix 1). Access to the electronic instrument for data collection was made available to participants through the Google Drive tool.

The application of the NASA TLX provides a subjective assessment of mental workload, namely, an assessment from the perspective of the person performing the task (self-assessment). This tool was chosen because of its ease of application, objectivity, and low cost of implementation [7].

The assessment of the participants’ mental workload was performed in 3 stages: assessment of weights for each dimension, assessment of rates for each dimension, and calculation of mental workload index (overload).

Initially, for the assessment of the weights for each dimension, 15 pairs of combinations between 6 assessment dimensions were presented to the participants (effort and physical demand, mental demand and effort, temporal demand and frustration, frustration and effort, performance and frustration, temporal demand and mental demand, temporal demand and effort, physical demand and temporal demand, effort and performance, frustration and mental demand, physical demand and frustration, performance and mental demand, mental demand and physical demand, performance and temporal demand, and performance and physical demand). At this stage, only 1 option should be selected in each pair, which should be the most prominent one during the proposed activity [23].

The frequencies assigned to each dimension were summed up and the results were expressed on a scale of 0-5. Then the rates assigned to each dimension were identified from the participants’ indication on a scale of 0-100 with 20 five-point equal intervals [23].

The weight of each dimension was multiplied by its respective rate, and the magnitude of mental workload was obtained. The magnitude values of the 6 dimensions were summed up, their results divided by 15, and the mental workload (overload) index was obtained [23].

The data were exported to electronic spreadsheets using Excel for Mac 2011 for data classification and analysis of the mental workload (overload) index. The dimensions comprising this mental workload were analyzed by descriptive (mean, minimum, maximum, standard deviation, and variance) and inferential (Student t test, analysis of variance [ANOVA] and Bonferroni) statistics. A significance level of *P*<.05 for a 95% confidence interval was used in this study.

## Results

### Students’ and Specialist Nurses’ Characteristics

The sample (n=75) comprised young students between 19 and 32 years of age (22.65 ± 3.12 years). Most were women (92%, 69/75) and exclusively dedicated to studying (79%, 59/75); only 8% (n=6) were working as nursing technicians.

Regarding the semester of the students (n=75) at the time of data collection, the following distribution was observed: 1% (n=1) in the second semester; 27% (n=20) in the third semester; 17% (n=13) in the fourth semester; 9% (n=7) in the fifth semester; 9% (n=7) in the sixth semester; 12% (n=9) in seventh semester, and 24% (n=18) in the eighth semester.

As for the use of the Internet in their personal and academic environments (multiple-choice questions), 131 answers were obtained, distributed as follows: 46% (60/131) accessed the Internet on their laptops; 34% (45/131) on mobile phones; 13% (17/131) on desktops, and 7% (9/131) on tablets.

The results draw attention to the issue of connectivity from small-sized mobile devices, thereby confirming the global trend of popularization of these technologies among young people [24].

Regarding the sociodemographic characteristics of specialist nurses (n=5), 100% (n=5) were female, 80% (n=4) had a master’s degree, and 20% (n=1) had a doctorate. As for the area of experience, the participants were distributed in the following areas: education and/or providing care for pain (40%, 2/5), nursing informatics (40%, 2/5), and medical surgical (20%, 1/5). All used mobile technologies and the Internet for personal and academic reasons every day.

### Mental Workload Assessment

The application of NASA TLX enabled identification of the rates, weights, and magnitudes assigned to 6 assessment dimensions, which were used to calculate the overload. Tables 1 and 2 show the results obtained from the assessment performed by students and specialists, as well as the mean overload for each group, respectively.

**Table 1:** Rate, weight, magnitude and mean overload^a^ of students (n=75).

Dimensions	Rate		Weight		Magnitude	
	Mean	SD	Mean	SD	Mean	SD
Mental demand	57.20	22.27	3.80	.92	217.27	110.41
Physical demand	29.27	27.91	1.08	.93	30.47	42.83
Temporal demand	37.67	24.14	3.08	1.47	122.27	102.26
Performance	58.47	24.19	3.55	1.21	216.73	121.65
Effort	40.73	23.24	2.69	.84	112.13	72.75
Frustration	30.60	27.80	0.80	1.15	36.93	71.89

^a^Mean overload of the students is 47.87.

**Table 2:** Rate, weight, magnitude, and mean overload of specialists (n=5).^a^

Dimensions	Rate		Weight		Magnitude	
	Mean	SD	Mean	SD	Mean	SD
Mental demand	51	29.45	3.40	.89	175	110.79
Physical demand	10	8.66	.40	.55	2	2.74
Temporal demand	30	34.10	2.60	1.82	89	112.05
Performance	73	28.85	4	1	294	134.69
Effort	49	34.53	2.20	1.30	121	129.05
Frustration	21	19.49	2.40	1.82	72	80.44

^a^Mean overload of the specialists is 50.20.

**Figure 3:**
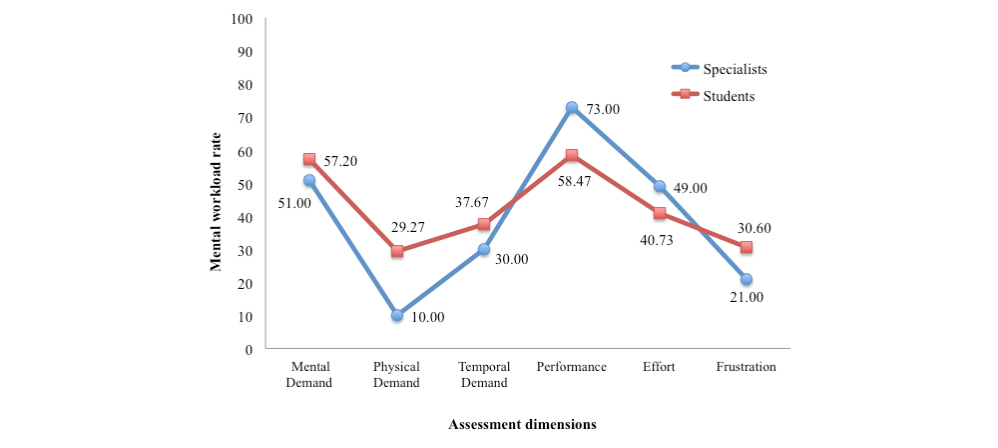
Mean rates of students (n=75) and specialists (n=5) for the NASA TLX dimensions.

Students and specialists obtained the highest means in these respective dimensions: “Performance” (58.47 ± 24.19, 73 ± 28.85) and “Mental Demand” (57.10 ± 22.27, 51 ± 29.45). It should be noted that the dimension “Physical Demand” (29.27 ± 27.91, 10 ± 8.66) was identified as the one that least contributed to mental workload during the educational intervention. In general, the distribution of rates assigned by the participants for each dimension had the same behavior (Figure 3).

The repeated measures one-way ANOVA was used to identify statistically significant differences in the mean values of the 6 dimensions of mental workload for the group of students (Table 3).

**Table 3:** Analysis of variance between means of assessments of the 6 dimensions of NASA TLX by students.

Assessment sources	Sum of squares	Degrees of freedom	Mean squares	Critical factor	*P* value
Factor 1 (6 dimensions)	61.053	5	13.570	29.45	<.001
Error	153.417	333	461	—	—

To identify the significant variables according to the ANOVA test, the Bonferroni test was applied, with no statistically significant difference in the mean values of the following pairs: “Mental Demand” and “Performance,” “Physical Demand” and “Temporal Demand,” “Physical demand” and “Frustration,” “Temporal Demand” and “Effort,” and also “Temporal Demand” and “Frustration.” For the other possible comparisons, significant differences between means were identified (Table 4).

**Table 4:** Comparison between the means of mental workload in 6 dimensions analyzed for students (n=75).^a^

Dimensions	Mean	95% Confidence interval
Mental demand	57.20^b^	52.07-62.35
Physical demand	29.27^c^	22.85-35.69
Temporal demand	37.67^cd^	32.11-43.22
Performance	58.47^b^	52.90-64.03
Effort	40.73^d^	35.38-46.09
Frustration	30.60^c^	24.20-37.00

^a^Equal lowercase letters do not statistically differ according to the Bonferroni test.

**Figure 4:**
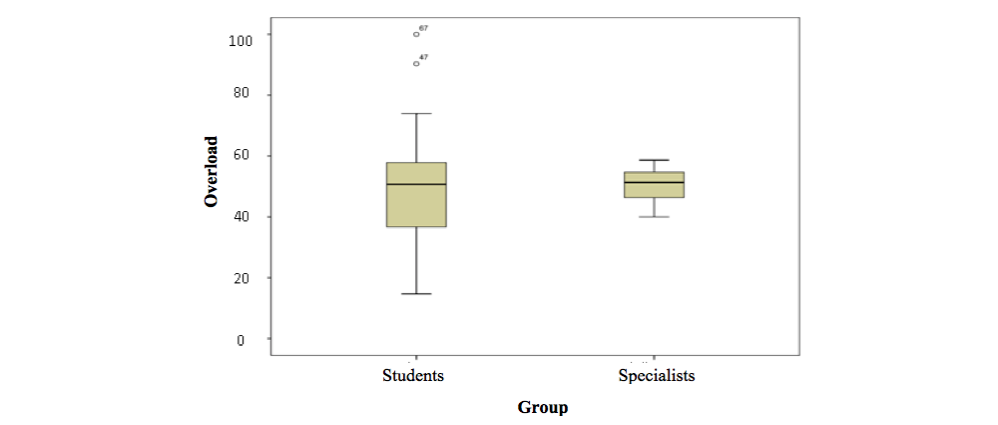
Box plot of the students’ (n=75) and specialists’ (n=5) overload means.

The ANOVA test could not be performed for comparison of means of assessment of the instrument dimensions due to the small number of specialist nurses. With regard to the mental workload index (overload), the specialists’ mean was higher (50.20 ± 7.28) when compared with the students’ mean (47.87 ± 16.85). Among the students, 2 outliers were identified (S47 and S67), which had high overload means (90.33 and 100), moving away from the means of most students (Figure 4).

## Discussion

### Preliminary Findings

The increasing inclusion of VLOs and mobile devices for teaching and learning in nursing has also been raising concerns about the assessment of mental workload that these resources can cause in their users. The assessment of these factors becomes essential to develop new directions for technological productions using these resources.

The analysis of the perceived mental workload from a given task is complex and personal, because it includes both specific characteristics of the task and the effort required for its development, which also has a direct relationship with the individual’s personal factors such as motivation, skills [3].

From the application of the NASA TLX instrument after an educational intervention using a VLO, we present the discussion of our results, starting with the sample characterization, followed by an analysis of individual assessment rates of the 6 dimensions of the instrument and the overload of students and specialists.

During the analysis of the students’ sociodemographic characteristics, it was found that although UFSC provides free Wi-Fi networks for students, students report that they most frequently access the Internet at their own homes 63% (70/111). This finding can be explained by the fact that the students are more focused on actual classes at the university, using other spaces to complement their online learning.

The fact that 7% (8/111) of the students access the Internet regularly during commuting also drew attention, confirming the global trend of mobility of individuals made possible by the use of mobile devices. Currently, technologies are very popular, especially for providing easy access to information, regardless of time or space [25].

Regarding rates, the 6 dimensions were similarly assessed by both students and specialists, with the dimensions “Performance” and “Mental Demand” standing out, as these had the highest assessment means.

The dimension “Performance” aimed at measuring how satisfied participants felt with their own level of performance for the assessment of pain using the m-OVADor. It had the highest rate of assessment, both among students (58.47 ± 24.19) and specialists (73 ± 28.85).

The assessment indicates a positive contribution of technology to learning acute pain assessment using mobile devices, suggesting it as a viable process to be used in the teaching-learning process in nursing, comparable to results of previous studies [7-9].

By contrast, “Mental Demand” had the second highest rate, both among students (57.20 ± 22.27) and specialists (51 ± 29.45), indicating that the educational intervention through m-OVADor demanded a greater mental workload, especially regarding the amount of mental activity for execution (think, decide, remember, look, and search).

This may be associated with the fact that the simulation of pain assessment in clinical scenarios requires constant involvement and judgment by the individuals, and may also be due to accessing content from mobile devices.

By contrast, “Physical Demand,” defined as the amount of physical activity that the online educational intervention demands from the individual, was indicated by students (29.27 ± 27.91) and specialists (10 ± 8.66) as the dimension that contributed least to the mental workload, just as reported in a previous nursing study [18].

For some authors [26], this result is justified by the low physical demand required for the handling of small mobile devices, which are very popular nowadays. The result is justified by both the familiarity of these individuals with access to the Internet, and the use of their own mobile devices in their academic and personal daily life, which would facilitate interaction with them. In addition, there is the project presentation, which sought to simplify the presentation layout of m-OVADor, thereby facilitating navigation through clinical scenarios.

The presence of 2 outliers (S47 and S67), which were distanced from the mean of most participants, with high levels of overload from the online educational intervention, can be explained by the individual perception of these individuals at the time of data collection, occurring at the end of the school year, when all students had examinations and term papers.

It must be considered that the assessment of mental workload is considered a complex and personal function, which also involves the specific characteristics of the task, the effort for its performance, and the direct relationship between motivation, emotional state, skills, among others [7].

Still, workload can be influenced by the nature of the task, especially when considering that the educational intervention with m-OVADor included access to 3 different clinical scenarios, in which there were a diversity of resources (animation, short texts with definitions and/or descriptions, assessment exercises, and also the clinical judgment to determine nursing diagnoses and interventions), required for the assessment of each presented context.

One should also consider that mental workload generated from VLOs can relate to factors such as the complexity of the individual, resources used for the development of the activity, and factors unrelated to content, such as the multimedia resources used [27-29].

In this sense, one should also reflect on increasing access to mobile devices with Internet access, and the potential of these technologies when applied in the teaching-learning process in nursing.

Arguably these technologies have become popular, especially for the flexibility that allows users to quickly access information at any place or time, to expand their potential to transform individuals’ attitudes and behaviors [30,31].

Specifically in the context of this study, an assessment of mental workload has indicated that the use of these technologies is feasible in higher education in nursing, and can influence the way students build their knowledge, establishing an innovative process for teaching/learning.

### Conclusions

The NASA TLX instrument demonstrated applicability for the assessment of mental workload from the use of m-OVADor. The results enabled analysis of the sources related to students’ and specialists’ mental workload from the use of a VLO accessed from small mobile devices. The analysis revealed that the dimension “Mental demand” obtained a greater weight both from the students’ and specialists’ perceptions, which is expected from technologies for online learning, due to the level of attention that they require. Although the study achieved the proposed aim, in no way did it intend to exhaust the possible approaches to the theme. The development of other studies to explore the theme from other perspectives is recommended, to deepen our understanding of the effects of mental workload on learning outcomes and even when planning new strategies for mobile learning in nursing education as well as for validation of the instrument in Brazil.

## Appendix

Multimedia Appendix 1 Nasa Task Load Index Form.

Multimedia Appendix 2 m-OVADor cover page.

Multimedia Appendix 3 Clinical scenarios selection by m-OVADor.

## Abbreviations

ANOVA: analysis of variance
ICTs: information and communications technologies
m-OVADor: Mobile Virtual Learning Object for the Assessment of Acute Pain
NASA TLX: NASA Task Load Index
VLO: virtual learning object
UFSC: Universidade Federal de Santa Catarina
