# Supplementary figures and images for: Mobile Virtual Learning Object for the Assessment of Acute Pain as a Learning Tool to Assess Acute Pain in Nursing: An Analysis of the Mental Workload
Source: JMIR Med Educ. 2015 Nov 6;1(2):e15. doi: 10.2196/mededu.4958 (PMC5041357; doi:10.2196/mededu.4958)

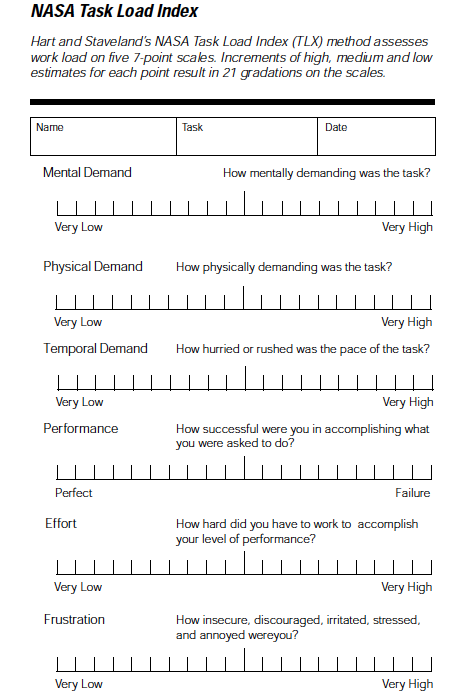

Supplement: Multimedia Appendix 1 [file mededu_v1i2e15_app1.png]

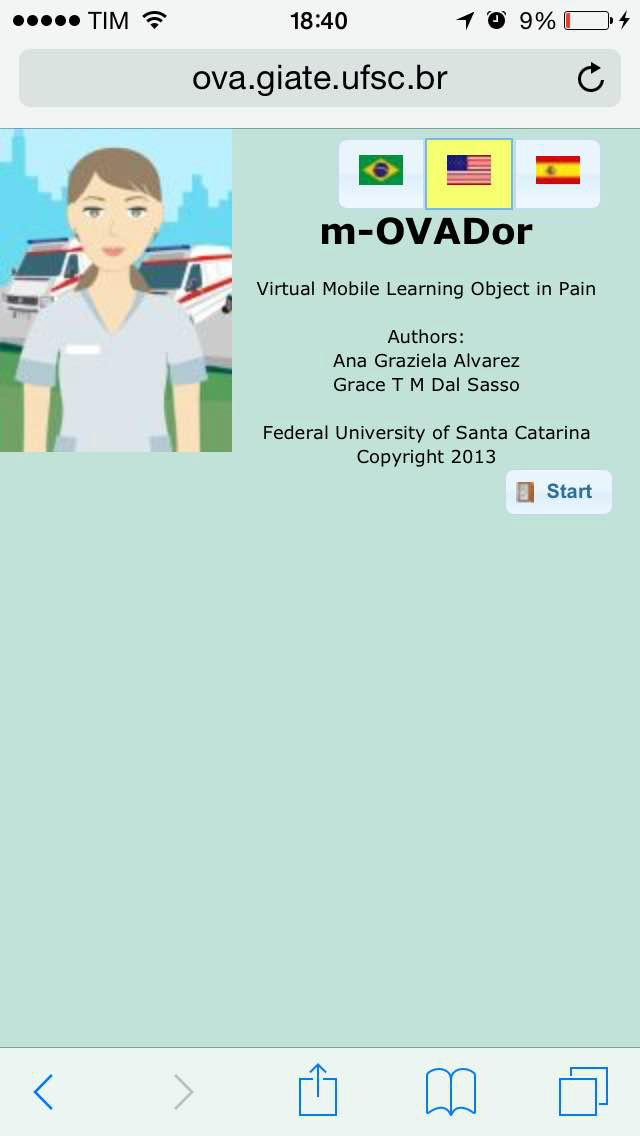

Supplement: Multimedia Appendix 2 [file mededu_v1i2e15_app2.png]

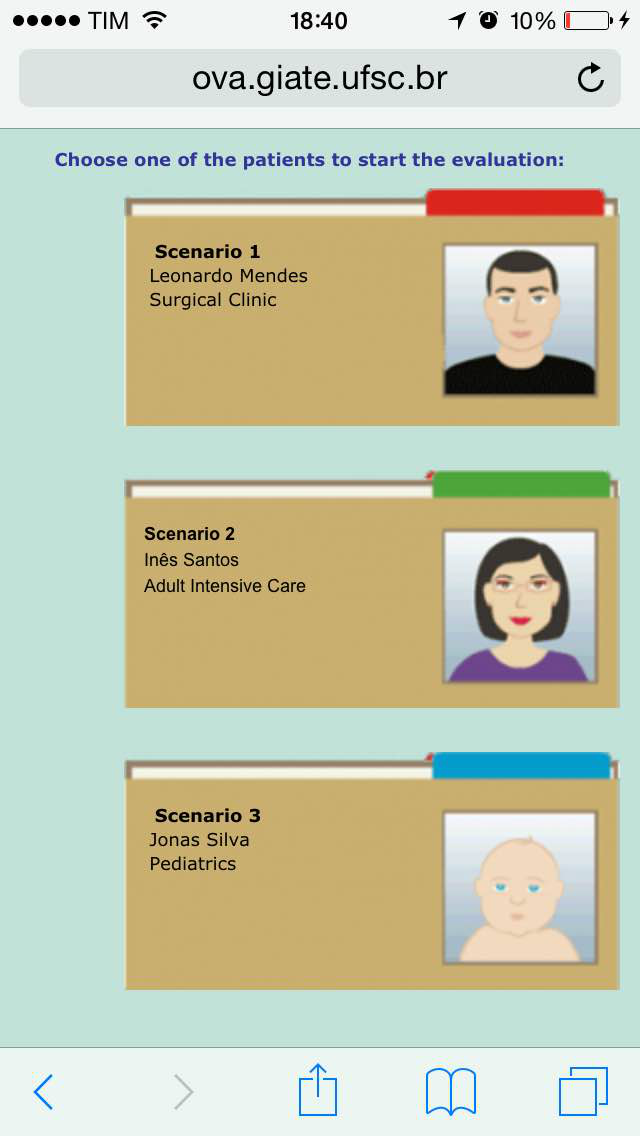

Supplement: Multimedia Appendix 3 [file mededu_v1i2e15_app3.png]
